# Supplementary material for: MFmap: A semi-supervised generative model matching cell lines to tumours and cancer subtypes
Source: PLoS One. 2021 Dec 16;16(12):e0261183. doi: 10.1371/journal.pone.0261183 (PMC8675718; doi:10.1371/journal.pone.0261183)
Supplement: S1 File — (PDF) [file pone.0261183.s001.pdf]

Supplementary materials for

MFmap: A semi-supervised generative model matching cell lines to tumours and cancer subtypes

Xiaoxiao Zhang[1,2], Maik Kschischo[1*]

1 Department of Mathematics and Technology, RheinAhrCampus, University of Applied Sciences Koblenz, Remagen, Germany
2 Department of Informatics, Technical University of Munich, Munich, Germany

* kschischo@rheinahrcampus.de

This PDF file includes extended method details.

TCGA bulk tumour subtype annotations

The TCGA bulk tumour subtype annotations were collected from literatures listed in Table 1

Table 1. Subtype labels for 10 TCGA bulk tumours annotated from literatures.

TCGA code	study name	reference
BRCA	Breast invasive carcinoma	[1]
COADREAD	Colon adenocarcinoma	[2]
ESCA	Esophageal carcinoma	[3]
HNSC	Head and neck squamous cell carcinoma	[4]
LUAD	Lung adenocarcinoma	[5]
LUSC	Lung squamous cell carcinoma	[6]
PAAD	Pancreatic adenocarcinoma	[7]
SKCM	Skin cutaneous melanoma	[8]
UCEC	Uterine corpus endometrial carcinoma	[9]
GBMLGG	Glioblastoma multiforme and lower grade glioma	[10]

Bulk tumor molecular data processing

Copy number, gene expression and mutation data from bulk tumours for 10 cancer types listed in Table 1 in the main text were obtained from Firehose Broad GDAC portal (http://gdac.broadinstitute.org/). Copy number $Log2$ ratio segment data genome were input into GISTIC2.0 (version 2.0.23) [11] to obtain thresholded CNV values. These data were dichotomised and stored in a sample by gene binary matrix, where 0 indicates a diploid copy number level and 1 indicates one of the following CNV levels: homozygous deletion, heterozygous deletion, low-level amplification, high-level amplification.

RSEM normalised gene expression data were preprocessed as follows: genes with missing values in more than 20% of the samples were excluded. For the remaining genes, missing values were imputed using `impute.knn` function from `impute` package [12]. The resulting gene expression values were transformed by adding 1.0 to each value and taking the

logarithm (pseudo-log-transformation).

Mutation data were downloaded as MAF files and synonymous mutations were excluded. The single-sample MAF files were further aggregated into a binary matrix M, with entries $(M)_{i,j} = 1$ indicating that there is a nonsynonymous mutation at j in patient i.

Cell line molecular data processing

Cell line data for the same cancer types in Table 1 in the main text were downloaded from the CCLE portal (https://portals.broadinstitute.org/ccle) and processed in the same way as bulk tumours, except that ensemble ids were mapped to gene symbols using human genome Genecode V19.

Molecular data normalisation

All molecular data input into MFmap neural network are normalised to the $0-1$ range.

Bulk tumor clinical data

Multiple subtype classification schemes for TCGA BRCA, COADREAD and GBMLGG exist and we decided to follow the most frequently used schemes. COADREAD has four consensus molecular subtypes CMS1-4 obtained from gene expression data [13]. GBMLGG [10] has seven subtypes (IDHmut-codel or Codel, G-CIMP-low, G-CIMP-high, Classic-like, Mesenchymal-like, LGm6-GBM, PA-like) which were derived from multi-modal data sets including mutation, methylation and gene expression patterns. BRCA subtypes (Basal, Her2, Luminal A, Luminal B, and Normal) are based on PAM50 (Prediction Analysis of Microarray using 50 gene set) [14] features derived from gene expression data and obtained from [1]. In our study, only Basal, Her2, Luminal A,

Luminal B samples were kept. The subtype annotations of all studied cancer types listed in Table 1 in the main text can be found in Table 1.

Cell line drug sensitivity data

CCLE cell line sample annotation data were obtained from CCLE portal and drug sensitivity data were downloaded from the Cancer Therapeutics Response Portal (CTRP [15], www.broadinstitute.org/ctrp).

Correcting for batch effects between bulk tumour and cell line gene expression

Batch effects between bulk tumours and cell lines were corrected by using the function `Combat` from the R package SVA [16]. The label bulk tumour or cell line was used as the covariate.

Handling class imbalance

Cancer subtype datasets especially GBMLGG are unbalanced, which is a major reason of overfitting for machine learning models. To overcome this issue, we applied Synthetic Minority Over-sampling Technique (SMOTE) [17] implemented in SmoteClassif function of UBL [18] R package to oversample the minority subtypes, creating an equal balance with majority subtypes.

Propagating copy number and mutation profiles on the protein-protein interaction network

Mutation profiles were mapped to human cancer network A curated by pyNBS [19] as protein-protein interaction network (PPI) source, which aggregates different interaction types.

The propagating function in pyNBS with optimal signal diffusion distance parameter setting $\alpha = 0.7$ was applied to perform network propagation, described by the iterative process $x_{t+1} = \alpha x_t A + (1 - \alpha)x_0$ with a closed solution $x_\infty = (1 - \alpha A)^{-1} x_0$. Here x_0 is the sample-by-gene matrix, A is the adjacency matrix of the PPI, and α is the parameter controlling the diffusion distance. The same approach was used for dichotomised copy number profiles. The smoothed copy number profiles and mutation profiles (sample by gene matrices) were combined into a single DNA-view matrix.

Pathway activity scores

Gene expression data for MsigDB [20] gene sets were input to ssGSEA [21], which outputs sample-wise pathway activity scores.

Biological annotation of latent representations

For a given componet z_k of the latent representation, we selected all pathways whose activities are significantly associated with z_k (association was measured using the information coefficient, FDR threshold of 5%). Some pathways are associated with more than one latent representation. To resolve these ambiguities, the Pearson correlation coefficients were estimated as well. The selection of pathways was further refined by using the fold change of z_k intensities between subtypes with the highest two latent representations and the lowest two latent representations. Here fold change and significance were estimated by a linear model implemented in the limma package [22], only those pathways with FDR adjusted p-value less than 0.05 were further kept for human review.

Generating MFmap visualisations

Let $Z = (z_{ij})$ be the $n \times h$-matrix of latent representations. The element z_{ij} is the value of the latent representations $j \in \{1, \ldots, h\}$ for patient $i \in \{1, \ldots, n\}$. The MFmap visualisation proceeds in three steps:

Step 1: Generate coordinates C_{1j}, C_{2j} of the MFmap prominent component nodes j by projecting the columns of matrix Z to 2-D space. The projection is chosen in order to preserve the distances between samples (Sammon projection). Delaunay triangulation on the 2-D coordinates is used to connect neighbouring nodes.

Step 2: Project samples onto the MFmap layout. The coordinates (S_{1i}, S_{2i}) of sample $i \in \{1, \ldots, n\}$ are given as

$$S_{li} = \frac{1}{\sum_{j=1}^{h} z_{ij}^{\alpha}} \sum_{j=1}^{h} C_{lj} z_{ij}^{\alpha}, \quad l = 1, 2 \tag{1}$$

where α is a tuneable hyper-parameter controlling the distance between nodes.

Step 3: Generate MFmap contour lines and background colours based on the estimated sample density of each subtype. The density is obtained from a Gaussian kernel density estimate on the coordinate lattice corresponding to the projected MFmap latent representations. The probability estimate for the subtype with the highest probability is then used for MFmap visualisation.

In-silico perturbation analysis

Let $\boldsymbol{z}_i^{(s)}$ denote the latent space representation of a tumour sample or cell line i with subtype s. An *in silico* latent sample $\tilde{\boldsymbol{z}}_i = \boldsymbol{z}_i^{(s)} + \boldsymbol{\delta}$ is obtained by adding the latent space perturbation $\boldsymbol{\delta}$. Then, the artificial data point $\tilde{\boldsymbol{x}}_i$ is sampled from the decoder $\tilde{\boldsymbol{x}}_i \sim p(\boldsymbol{x}|\tilde{\boldsymbol{z}}_i)$. For Fig 8 in the main text we

used the $s =$ G-CIMP-high as the subtype of the original data. The perturbation $\boldsymbol{\delta} = \bar{\boldsymbol{z}}^{(s)} - \bar{\boldsymbol{z}}^{(s')}$ with $s' =$ Mesenchymal-like was obtained as the difference between the mean latent space vectors of both subtypes.

Design of MFmap neural network

The MFmap encoder network

The encoder receives a gene expression profile $\boldsymbol{x}_{rna}$ and a propagated DNA alteration profile $\boldsymbol{x}_{dna}$ as input. Two hidden layers first encode the two input layers into two 1024-dimensional latent vectors. The second hidden layer then concatenates the two 1024-dimensional latent vectors and then is further encoded into 512-dimensional vector by the third hidden layer. The third hidden layer is fully connected to two output layers representing mean $\boldsymbol{\mu}$ and log-transformed variation $\log \boldsymbol{\sigma}^2$ of $q_\phi(\boldsymbol{z}|\boldsymbol{x})$. The dimension of the latent representation $\boldsymbol{z}$ is set as the number of subtypes of cancer. The encoder is given by the following equations:

$$\boldsymbol{x}^1_{e_{rna}} = \text{ReLU}(W^0_{e_{rna}} \cdot \boldsymbol{x}_{rna} + \boldsymbol{b}^1_{e_{rna}}) \tag{2}$$
$$\boldsymbol{x}^1_{e_{dna}} = \text{ReLU}(W^0_{e_{dna}} \cdot \boldsymbol{x}_{dna} + \boldsymbol{b}^1_{e_{dna}}) \tag{3}$$
$$\boldsymbol{x}^2_{e} = \text{ReLU}(W^1_{e} \cdot \boldsymbol{x}^1_{e_{rna}} \oplus \boldsymbol{x}^1_{e_{dna}} + \boldsymbol{b}^2_{e}) \tag{4}$$
$$\boldsymbol{x}^3_{e} = \text{ReLU}(W^2_{e} \cdot \boldsymbol{x}^2_{e} + \boldsymbol{b}^3_{e}) \tag{5}$$
$$\boldsymbol{\mu} = \text{ReLU}(W^\mu_{e} \cdot \boldsymbol{x}^3_{e} + \boldsymbol{b}^\mu) \tag{6}$$
$$\log(\boldsymbol{\sigma}^2) = \text{ReLU}(W^\sigma_{e} \cdot \boldsymbol{x}^3_{e} + \boldsymbol{b}^\sigma) \tag{7}$$

where $W^0_{e_{rna}} \in \mathbb{R}^{k_{rna} \times h^1_{rna}}$,$\boldsymbol{b}^1_{e_{rna}} \in \mathbb{R}^{1 \times h^1_{rna}}$, $W^0_{e_{dna}} \in \mathbb{R}^{k_{dna} \times h^1_{dna}}$,$\boldsymbol{b}^1_{e_{dna}} \in \mathbb{R}^{1 \times h^1_{dna}}$, $W^1_e \in \mathbb{R}^{h^1_{rna}+h^1_{dna} \times h^2}$,$\boldsymbol{b}^2_e \in \mathbb{R}^{1 \times h^2}$, $W^2_e \in \mathbb{R}^{h^2 \times h^3}$,$\boldsymbol{b}^3_e \in \mathbb{R}^{1 \times h^3}$, $W^\mu \in \mathbb{R}^{h^3 \times m}$,$\boldsymbol{b}^\mu \in \mathbb{R}^{1 \times m}$ $W^\sigma \in \mathbb{R}^{h^3 \times m}$,$\boldsymbol{b}^\sigma \in \mathbb{R}^{1 \times m}$ are trainable parameters of the encoder network. Rectified linear unit (ReLU) activation is defined as $\text{ReLu}(x) = \max(x, 0)$. $\oplus$ denotes concatenating two matrices.

The MFmap decoder network

The MFmap decoder layer structure is symmetric to the encoder with latent representations $\boldsymbol{z}$ as inputs and reconstructed data as outputs $\boldsymbol{x}'_{rna}$ and $\boldsymbol{x}'_{dna}$:

$$\boldsymbol{x}_d^1 = \text{ReLU}(W_d^0 \cdot z + \boldsymbol{b}_d^1) \tag{8}$$
$$\boldsymbol{x}_d^2 = \text{ReLU}(W_d^1 \cdot \boldsymbol{x}_d^1 + \boldsymbol{b}_d^2) \tag{9}$$
$$\boldsymbol{x}_{d_{rna}}^3 = \text{ReLU}(W_{d_{rna}}^2 \cdot \top^{h_{rna}^1}(\boldsymbol{x}_d^2) + \boldsymbol{b}_{d_{rna}}^3) \tag{10}$$
$$\boldsymbol{x}_{d_{dna}}^3 = \text{ReLU}(W_{d_{dna}}^2 \cdot \bot^{h_{dna}^1}(\boldsymbol{x}_d^2) + \boldsymbol{b}_{d_{dna}}^3) \tag{11}$$
$$\boldsymbol{x}'_{rna} = \sigma(W_{rna}^o \cdot \boldsymbol{x}_{d_{rna}}^3 + \boldsymbol{b}_{rna}^o) \tag{12}$$
$$\boldsymbol{x}'_{dna} = \sigma(W_{dna}^o \cdot \boldsymbol{x}_{d_{dna}}^3 + \boldsymbol{b}_{dna}^o)\,. \tag{13}$$

Here, $W_d^0 \in \mathbb{R}^{m\times h^3}$,$\boldsymbol{b}_d^1 \in \mathbb{R}^{1\times h^3}$, $W_d^1 \in \mathbb{R}^{h^3\times h^2}$,$\boldsymbol{b}_d^2 \in \mathbb{R}^{1\times h^2}$, $W_{d_{rna}}^2 \in \mathbb{R}^{h^2\times h_{rna}^1}$,$\boldsymbol{b}_d^2 \in \mathbb{R}^{1\times h_{rna}^1}$, $W_{d_{dna}}^2 \in \mathbb{R}^{h^2\times h_{dna}^1}$,$\boldsymbol{b}_d^2 \in \mathbb{R}^{1\times h_{dna}^1}$, $W_{rna}^o \in \mathbb{R}^{h_{rna}^1\times k_{rna}}$,$\boldsymbol{b}_{rna}^o \in \mathbb{R}^{1\times h_{rna}^1}$, $W_{dna}^o \in \mathbb{R}^{h_{dna}^1\times k_{dna}}$,$\boldsymbol{b}_{dna}^o \in \mathbb{R}^{1\times h_{dna}^1}$ are trainable parameters of the decoder network. The sigmoid activation is defined as $\sigma(x) = \frac{1}{1+e^{-x}}$. The subsetting operation of the top and bottom j elements of x is denoted by $\top^j(x)$ and $\bot^j(x)$, respectively.

The MFmap classifer network

The classifier serves as a regulariser controlling the capability to learn a latent representation that is cancer subtype relevant. Since cancer subtypes are clinically and biologically meaningful, a higher classification accuracy will encourage the neural network to extract features essential to patients stratification and are more interpretable. The classifier is a neural network with three fully connected layers and takes the expectation $\boldsymbol{\mu}$ (see Eq (6)) of the distribution $p(\boldsymbol{z}\,|\boldsymbol{x})$ with $\boldsymbol{x} = (\boldsymbol{x}_{rna}, \boldsymbol{x}_{dna})$ as input and outputs a probability for

each of the h subtypes:

$$\boldsymbol{x}_c^1 = \text{ReLU}(W_c^0 \cdot \boldsymbol{\mu} + \boldsymbol{b}_c^1) \tag{14}$$
$$\boldsymbol{x}_c^2 = \text{ReLU}(W_c^1 \cdot \boldsymbol{x}_c^1 + \boldsymbol{b}_c^2) \tag{15}$$
$$\boldsymbol{x}_o = \text{softMax}(W_c^o \cdot \boldsymbol{x}_c^2 + \boldsymbol{b}_c^o), \tag{16}$$

where $W_c^0 \in \mathbb{R}^{m \times h_c^1}$,$\boldsymbol{b}_c^1 \in \mathbb{R}^{1 \times h_c^1}$, $W_c^1 \in \mathbb{R}^{h_c^1 \times h_c^2}$,$\boldsymbol{b}_c^2 \in \mathbb{R}^{1 \times h_c^2}$, $W_c^o \in \mathbb{R}^{h_c^2 \times s}$,$\boldsymbol{b}_c^o \in \mathbb{R}^{1 \times s}$ are trainable parameters of the classifier. The softmax activation is defined as $\text{softMax}(x, y = c) = \frac{e^{x_C}}{\sum_{j=1}^{C} e^{x_j}}$, given the inputs and label pair $(x, y = c)$.

Details of the MFmap hidden layers

Each hidden layer is a block containing a fully connected layer, a batch normalisation layer and an activation.

Details of the MFmap loss function

The MFmap loss function in Eq (10) in the main text for the specific distributional assumptions can be rewritten as

$$\begin{cases} \mathcal{S}(\boldsymbol{x}, y) = \sum_{v \in \{rna,dna\}} \mathcal{L}_{\text{recon}}(\boldsymbol{x}_v, \hat{\boldsymbol{x}}_v) + \mathcal{D}_{KL}(\mathcal{N}(\boldsymbol{\mu}, \boldsymbol{\sigma}) \parallel \mathcal{N}(\mathbf{0}, \boldsymbol{I})) + \mathcal{L}_{\text{CE}}(y, \hat{y}) + \mathcal{H}(\hat{y}), & (\boldsymbol{x}, y) \in \mathcal{D}_{tu}, \\ \mathcal{U}(\boldsymbol{x}) = \sum_{v \in \{rna,dna\}} \mathcal{L}_{\text{recon}}(\boldsymbol{x}_v, \hat{\boldsymbol{x}}_v) + \mathcal{D}_{KL}(\mathcal{N}(\boldsymbol{\mu}, \boldsymbol{\sigma}) \parallel \mathcal{N}(\mathbf{0}, \boldsymbol{I})) + \mathcal{H}(\hat{y}), & \boldsymbol{x} \in \mathcal{D}_{cl}. \end{cases} \tag{17}$$

In Eq (17), $\boldsymbol{\mu}$, $\boldsymbol{\sigma}$ are estimated parameters of MFmap encoder. $\hat{y}$ denotes the subtype label probability predicted by the MFmap classifier and $\mathcal{L}_{\text{recon}}$, $\mathcal{D}_{KL}$, $\mathcal{L}_{\text{CE}}$, and $\mathcal{H}$ denote the reconstruction loss, KL divergence, cross entropy loss and entropy, respectively. We next detail each term.

The reconstruction loss $\mathcal{L}_{\text{recon}}$ is quantified by the binary cross entropy loss between input data $\boldsymbol{x}$ and reconstructed data $\hat{\boldsymbol{x}}$:

$$\mathcal{L}_{\text{recon}}(\boldsymbol{x}, \hat{\boldsymbol{x}}) = -\sum_{i=1}^{d} \boldsymbol{x}_i \log(\hat{\boldsymbol{x}}_i) + (1 - \boldsymbol{x}_i) \log(1 - \hat{\boldsymbol{x}}_i). \tag{18}$$

The classification loss for sample i is implemented as a cross entropy loss

$$\mathcal{L}_{\mathrm{CE}}(y^{(i)}, \hat{y}^{(i)}) = -\sum_{k=1}^{h} y_k^{(i)} \log(\hat{y}_k^{(i)}). \tag{19}$$

The entropy of sample i is implemented as

$$\mathcal{H}(\hat{y}^{(i)}) = -\sum_{k=1}^{h} \hat{y}_k^{(i)} \log(\hat{y}_k^{(i)}). \tag{20}$$

In Eq (19) and Eq (20) $\hat{y}_k^{(i)} = \mathrm{softMax}_k(\boldsymbol{c}(\boldsymbol{x}^{(i)};\theta))$, where $\boldsymbol{c}(\cdot) = (c_1(\cdot), \ldots, c_h(\cdot))$ is the classification model parametrised by θ, and $\mathrm{softMax}_k$ is the softmax function for subtype label k.

In fact, for bulk tumours the MFmap loss function can be viewed as a basic VAE loss plus an entropy regularised classification loss which is equivalent to a modified soft bootstrapping loss proposed by [23] for positive unlabelled learning, extended to multi-class case. It updates the prediction objective based on currently predicted subtype probability. Concretely, the modified soft bootstrapping loss for a pair of feature and subtype label $(\boldsymbol{x}^{(i)}, y^{(i)})$ is:

$$l_{sb}(\boldsymbol{x}^{(i)}, y^{(i)}; \theta) = \begin{cases} -\log(\mathrm{softMax}_1(\boldsymbol{c}(\boldsymbol{x}^{(i)};\theta))) - \sum_{k=1}^{h} \mathrm{softMax}_k(\boldsymbol{c}(\boldsymbol{x}^{(i)};\theta)) \log(\mathrm{softMax}_k(\boldsymbol{c}(\boldsymbol{x}^{(i)};\theta))), & y^{(i)} = 1, \\ \vdots & \\ -\log(\mathrm{softMax}_h(\boldsymbol{c}(\boldsymbol{x}^{(i)};\theta))) - \sum_{k=1}^{h} \mathrm{softMax}_k(\boldsymbol{c}(\boldsymbol{x}^{(i)};\theta)) \log(\mathrm{softMax}_k(\boldsymbol{c}(\boldsymbol{x}^{(i)};\theta))), & y^{(i)} = h. \end{cases} \tag{21}$$

Gradients of the MFmap loss function can be computed with the reparametrisation trick [24], which involves sampling a random vector $\boldsymbol{\epsilon} \sim \mathcal{N}(\boldsymbol{\epsilon}|\boldsymbol{\epsilon}; \mathbf{0}, \boldsymbol{I})$ and transforming to

$$\boldsymbol{z} = \boldsymbol{\mu}(\boldsymbol{x}) + \boldsymbol{\sigma}(\boldsymbol{x}) \odot \boldsymbol{\epsilon}. \tag{22}$$

This ensures that $\boldsymbol{z} \sim q(\boldsymbol{z}|\boldsymbol{x}) = \mathcal{N}(\boldsymbol{z}|\boldsymbol{\mu}(\boldsymbol{x}), \mathrm{diag}(\boldsymbol{\sigma})^2)$.

Details of the MFmap implementation and training process

The MFmap neural network was implemented using PyTorch (version 1.5.0) and trained on two NVIDIA Tesla V100 SXM2 GPUs (each has memory of 32 gigabytes) using the Adam optimiser [25]. The preprocessed data were randomly split into training (90%) and test (10%) sets. Hyperopt [26] was used to search the best model avoiding overfitting by selecting optimal hyper-parameters yielding the minimum total loss divergence between training and validation datasets (ratio training/validation data 9/10). The searching space for hyper-parameter selection is:

- dimensions of first DNA or RNA encoder hidden layer $\{4096, 2048, 1024\}$
- dimensions of second DNA or RNA encoder hidden layer $\{1024, 512, 256\}$
- dimensions of third DNA or RNA encoder hidden layer $\{256, 128, 64\}$
- dimensions of first classifier hidden layer $\{512, 256, 128, 64\}$
- dimensions of second classifier hidden layer $\{128, 64, 32\}$
- batch size $\{64, 32\}$
- learning rate $\{10^{-4}, 10^{-3}, 10^{-2}\}$

The optimised setting found from the minimal validation loss is: learning rate as 10^{-3}; batch size as 32; first, second and third encoder hidden layer dimension as $1024, 512, 256$ respectively; first, second classifier hidden layer dimension as $128, 64$ respectively. These hyper-parameters were used for all cancer types in Table 1 in the main text.

Derivation of the unsupervised and supervised ELBO

We first derive the ELBO for cell line data using Jensen's inequality:

$$\begin{aligned}
\log p(\boldsymbol{x}) &= \log\left(\sum_y \int d\boldsymbol{z}\, q(\boldsymbol{z}|\boldsymbol{x}) \frac{p(\boldsymbol{x}, y, \boldsymbol{z})}{q(\boldsymbol{z}|\boldsymbol{x})}\right) \\
&= \log\left(E_{q(\boldsymbol{z}|\boldsymbol{x})p(y|\boldsymbol{z})}\left[\frac{p(\boldsymbol{x}|\boldsymbol{z})p(\boldsymbol{z})}{q(\boldsymbol{z}|\boldsymbol{x})}\right]\right) \\
&\geq E_{q(\boldsymbol{z}|\boldsymbol{x})p(y|\boldsymbol{z})}\left[\frac{p(\boldsymbol{x}|\boldsymbol{z})p(\boldsymbol{z})}{q(\boldsymbol{z}|\boldsymbol{x})}\right] \\
&= E_{q(\boldsymbol{z}|\boldsymbol{x})}\left[\log p(\boldsymbol{x}|\boldsymbol{z})\right] - D_{KL}\left(q(\boldsymbol{z}|\boldsymbol{x})||p(\boldsymbol{z})\right). \qquad (23)
\end{aligned}$$

Here, we have used the conditional independence assumption Eq (3c) in the main text and $p(y|\boldsymbol{z}) = q(y|\boldsymbol{z})$. Similarly, for the labelled examples (bulk tumor samples), we can derive the ELBO for the log-likelihood as

$$\begin{aligned}
\log p(\boldsymbol{x}, y) &= \log\left(\int d\boldsymbol{z}\, q(\boldsymbol{z}|\boldsymbol{x}) \frac{p(\boldsymbol{x}, y, \boldsymbol{z})}{q(\boldsymbol{z}|\boldsymbol{x})}\right) \\
&\geq E_{q(\boldsymbol{z}|\boldsymbol{x})}\left[\log\left(\frac{p(\boldsymbol{x}|\boldsymbol{z})p(y|\boldsymbol{z})p(\boldsymbol{z})}{q(\boldsymbol{z}|\boldsymbol{x})}\right)\right] \\
&= E_{q(\boldsymbol{z}|\boldsymbol{x})}\left[\log p(\boldsymbol{x}|\boldsymbol{z})\right] - D_{KL}\left(q(\boldsymbol{z}|\boldsymbol{x})||p(\boldsymbol{z})\right) \\
&\quad + E_{q(\boldsymbol{z}|\boldsymbol{x})}\left[\log p(y|\boldsymbol{z})\right], \qquad (24)
\end{aligned}$$

where we have used the conditional independence of $\boldsymbol{x}$ and y given $\boldsymbol{z}$ in both the generative and the inference model.

References

1. Curtis C, Shah SP, Chin SF, Turashvili G, Rueda OM, Dunning MJ, et al. The genomic and transcriptomic architecture of 2,000 breast tumours reveals novel subgroups. Nature. 2012;486(7403):346–352. doi:10.1038/nature10983.

2. Guinney J, Dienstmann R, Wang X, de Reyniès A, Schlicker A, Soneson C, et al. The consensus molecular subtypes of colorectal cancer. Nature Medicine. 2015;21(11):1350–1356. doi:10.1038/nm.3967.

3. Kim J, Bowlby R, Mungall AJ, Robertson AG, Odze RD, Cherniack AD, et al. Integrated genomic characterization of oesophageal carcinoma. Nature. 2017;541(7636):169–175. doi:10.1038/nature20805.

4. Network CGA. Comprehensive genomic characterization of head and neck squamous cell carcinomas. Nature. 2015;517(7536):576–582. doi:10.1038/nature14129.

5. Collisson EA, Campbell JD, Brooks AN, Berger AH, Lee W, Chmielecki J, et al. Comprehensive molecular profiling of lung adenocarcinoma. Nature. 2014;511(7511):543–550. doi:10.1038/nature13385.

6. Hammerman PS, Lawrence MS, Voet D, Jing R, Cibulskis K, Sivachenko A, et al. Comprehensive genomic characterization of squamous cell lung cancers. Nature. 2012;489(7417):519–525. doi:10.1038/nature11404.

7. Moffitt RA, Marayati R, Flate EL, Volmar KE, Loeza SGH, Hoadley KA, et al. Virtual microdissection identifies distinct tumor- and stroma-specific subtypes of pancreatic ductal adenocarcinoma. Nature Genetics. 2015;47(10):1168–1178. doi:10.1038/ng.3398.

8. Akbani R, Akdemir KC, Aksoy BA, Albert M, Ally A, Amin SB, et al. Genomic Classification of Cutaneous Melanoma. Cell. 2015;161(7):1681–1696. doi:https://doi.org/10.1016/j.cell.2015.05.044.

9. Levine DA, Getz G, Gabriel SB, Cibulskis K, Lander E, Sivachenko A, et al. Integrated genomic characterization of endometrial carcinoma. Nature. 2013;497(7447):67–73. doi:10.1038/nature12113.

10. Ceccarelli M, Barthel FP, Malta TM, Sabedot TS, Salama SR, Murray BA, et al. Molecular Profiling Reveals Biologically Discrete Subsets and Pathways of Progression in Diffuse Glioma. Cell. 2016;164(3):550–563. doi:https://doi.org/10.1016/j.cell.2015.12.028.

11. Mermel CH, Schumacher SE, Hill B, Meyerson ML, Beroukhim R, Getz G. GISTIC2.0 facilitates sensitive and confident localization of the targets of focal somatic copy-number alteration in human cancers. Genome Biology. 2011;12(4):R41. doi:10.1186/gb-2011-12-4-r41.

12. Janssen KJM, Donders ART, Harrell FE, Vergouwe Y, Chen Q, Grobbee DE, et al. Missing covariate data in medical research: To impute is better than to ignore. Journal of Clinical Epidemiology. 2010;63(7):721–727. doi:https://doi.org/10.1016/j.jclinepi.2009.12.008.

13. Guinney J, Dienstmann R, Wang X, de Reyniès A, Schlicker A, Soneson C, et al. The consensus molecular subtypes of colorectal cancer. Nature Medicine. 2015;21(11):1350–1356. doi:10.1038/nm.3967.

14. Parker JS, Mullins M, Cheang MCU, Leung S, Voduc D, Vickery T, et al. Supervised Risk Predictor of Breast Cancer Based on Intrinsic Subtypes. Journal of

Clinical Oncology. 2009;27(8):1160–1167. doi:10.1200/JCO.2008.18.1370.

15. Seashore-Ludlow B, Rees MG, Cheah JH, Cokol M, Price EV, Coletti ME, et al. Harnessing Connectivity in a Large-Scale Small-Molecule Sensitivity Dataset. Cancer Discovery. 2015;5(11):1210. doi:10.1158/2159-8290.CD-15-0235.

16. Leek JT, Johnson WE, Parker HS, Jaffe AE, Storey JD. The sva package for removing batch effects and other unwanted variation in high-throughput experiments. Bioinformatics. 2012;28(6):882–883. doi:10.1093/bioinformatics/bts034.

17. Chawla NV, Bowyer KW, Hall LO, Kegelmeyer WP. SMOTE: Synthetic Minority Over-sampling Technique. Journal of Artificial Intelligence Research. 2002;16:321–357. doi:10.1613/jair.953.

18. Branco P, Ribeiro RP, Torgo L. UBL: an R package for Utility-based Learning. arXiv:160408079 [Preprint]. 2016;doi:Available from:https://arxiv.org/pdf/1604.08079.pdf.

19. Huang JK, Jia T, Carlin DE, Ideker T. pyNBS: a Python implementation for network-based stratification of tumor mutations. Bioinformatics. 2018;34(16):2859–2861. doi:10.1093/bioinformatics/bty186.

20. Liberzon A, Subramanian A, Pinchback R, Thorvaldsdóttir H, Tamayo P, Mesirov JP. Molecular signatures database (MSigDB) 3.0. Bioinformatics. 2011;27(12):1739–1740. doi:10.1093/bioinformatics/btr260.

21. Hänzelmann S, Castelo R, Guinney J. GSVA: gene set variation analysis for microarray and RNA-Seq data.

BMC Bioinformatics. 2013;14(1):7. doi:10.1186/1471-2105-14-7.

22. Ritchie ME, Phipson B, Wu D, Hu Y, Law CW, Shi W, et al. limma powers differential expression analyses for RNA-sequencing and microarray studies. Nucleic acids research. 2015;43(7):e47–e47. doi:10.1093/nar/gkv007.

23. Reed S, Lee H, Anguelov D, Szegedy C, Erhan D, Rabinovich A. Training Deep Neural Networks on Noisy Labels with Bootstrapping. arXiv:14126596[Preprint]. 2015;doi:Available from:https://arxiv.org/pdf/1412.6596.pdf.

24. Kingma DP, Welling M. Auto-Encoding Variational Bayes. arXiv:13126114 [Preprint]. 2013;doi:Available from: https://arxiv.org/pdf/1312.6114.pdf.

25. Kingma DP, Ba J. Adam: A Method for Stochastic Optimization. arXiv:14126980 [Preprint]. 2014;doi:Available from: https://arxiv.org/pdf/1412.6980.pdf.

26. Bergstra J, Komer B, Eliasmith C, Yamins D, Cox DD. Hyperopt: a Python library for model selection and hyperparameter optimization. Computational Science & Discovery. 2015;8(1):014008. doi:10.1088/1749-4699/8/1/014008.
