# Supplementary material for: MFmap: A semi-supervised generative model matching cell lines to tumours and cancer subtypes
Source: PLoS One. 2021 Dec 16;16(12):e0261183. doi: 10.1371/journal.pone.0261183 (PMC8675718; doi:10.1371/journal.pone.0261183)
Supplement: S2 File — (PDF) [file pone.0261183.s002.pdf]

Supplementary materials for

MFmap: A semi-supervised generative model matching cell lines to tumours and cancer subtypes

Xiaoxiao Zhang[1,2], Maik Kschischo[1*]

1 Department of Mathematics and Technology, RheinAhrCampus, University of Applied Sciences Koblenz, Remagen, Germany
2 Department of Informatics, Technical University of Munich, Munich, Germany

* kschischo@rheinahrcampus.de

This PDF file includes further evaluation of MFmap performance.

Further evaluation of the MFmap classification performance

Table 1. MFmap subtype classification performance estimated for unseen tumour samples. Here, 10% of the bulk tumour data were randomly selected as an independent test set. The dimensions of the latent representations are set to the number of cancer subtypes.

accuracy	precision	recall	F_1 score	organ
0.94	0.94	0.94	0.94	BRCA
0.98	0.99	0.98	0.98	COADREAD
1.00	1.00	1.00	1.00	ESCA
1.00	1.00	1.00	1.00	GBMLGG
0.94	0.95	0.94	0.94	HNSC
0.96	0.96	0.96	0.96	LUAD
1.00	1.00	1.00	1.00	LUSC
0.94	0.94	0.94	0.94	PAAD
1.00	1.00	1.00	1.00	SKCM
0.95	0.96	0.95	0.95	UCEC

Table 2. MFmap subtype classification performance estimated for unseen tumour samples. Here, 20% of the bulk tumour data were randomly selected as an independent test set. The dimensions of the latent representations are set to 100.

accuracy	precision	recall	F_1 score	organ
0.96	0.96	0.96	0.96	BRCA
0.95	0.95	0.95	0.95	COADREAD
1.00	1.00	1.00	1.00	ESCA
0.99	0.99	0.99	0.99	GBMLGG
0.91	0.91	0.91	0.91	HNSC
0.92	0.92	0.92	0.92	LUAD
0.92	0.92	0.92	0.92	LUSC
0.84	0.85	0.84	0.84	PAAD
0.98	0.98	0.98	0.98	SKCM
0.98	0.98	0.98	0.98	UCEC

Evaluating the overlap between a reference model and query data

To explore the potential use of MFmap in a transfer learning setting [1–3], we investigated, how well a model trained on a larger data set performs as a reference model. We evaluated the overlap between the latent space distribution of the reference model and a query dataset using four different performance measures: the entropy of subtype mixing (ESM),

the adjusted random index (ARI), the normalised mutual information (NMI) and the average silhouette width (ASW), see [1] for details.

We evaluated these measurements in three different scenarios:

1. Train MFmap with TCGA bulk tumour data and use this as a reference model. Use the CCLE cell line data as query set.
2. Randomly split the union of TCGA bulk tumour data and CCLE cell line data into a training set and a test set in the ratio of $8:2$. Use the training set to train the reference model and the test set as a query set.
3. Compared to 2. add normally distributed noise (mean 0.3 and standard deviation 0.5) to the standardised features in the test (query) data set.

The last scenario was used to check the robustness against a distribution shift in the query data relative to the reference data.

The entropy of subtype mixing (ESM)

To evaluate the ESM, we used the latent representation of each sample in the query data and looked for the 15 nearest neighbours in the latent space, which are both tumour samples (with known subtypes) and from the reference data. The ESM is calculated as $ESM = -\sum_{i=1}^{K} p_i \log p_i$, where p_i is the frequency of the neighbours belonging to subtype i. Then we averaged the ESM over all query samples and rescaled it to the range between zero (best reference mapping) and one (worst reference mapping). The rescaling corresponds to choosing the base of the logarithm equal to the number of subtypes.

The adjusted random index (ARI) and the normalised mutual information (NMI)

To estimate the ARI and NMI, we trained a 15-nearest neighbour classifier using latent representations of the reference data. This trained classifier was used to predict the subtype of the query data, using their latent representation as an input. The predicted subtype labels were compared to the predictions of the MFmap classification layer. Both ARI and NMI range between zero (worst matching) and one (best matching). We used a scaled version of the classical ASW as in [1], which is given as $ASW = \frac{ASW^{classical}+1}{2}$. The best possible separation between clusters is given by $ARI = 1$. We used the ARI, NMI and ASW implementations in the Scikit-Learn python package [4].

Table 3. Query and reference data overlapping evaluation. Here query data are CCLE cell line data and reference data are TCGA bulk tumour data.

	**ESM**[a]	**ARI**[b]	**NMI**[c]	**ASW_R**[d]	**ASW_Q**[e]	**ASW**[f]
GBMLGG	0.01	1.0	1.0	0.92	0.89	0.91
BRCA	0.00	1.0	1.0	0.91	0.90	0.90
COADREAD	0.00	1.0	1.0	0.93	0.94	0.93
HNSC	0.00	1.0	1.0	0.89	0.92	0.89
LUAD	0.00	1.0	1.0	0.93	0.92	0.92
LUSC	0.00	1.0	1.0	0.92	0.94	0.92
SKCM	0.00	1.0	1.0	0.93	0.92	0.93
UCEC	0.00	1.0	1.0	0.94	0.95	0.93
ESCA	0.00	1.0	1.0	0.96	0.92	0.95
PAAD	0.00	1.0	1.0	0.95	0.96	0.95

[a] Entropy of subtype mixing.
[b] Adjusted random index.
[c] Normalised mutual information.
[d] Average silhouette width in reference data.
[e] Average silhouette width in query data.
[f] Average silhouette width in both reference and query data.

Table 4. Query and reference data overlapping evaluation in unseen test data. Here query data are 20% test data unseen in training and reference data are TCGA bulk tumour data.

	ESM[a]	ARI[b]	NMI[c]	ASW_R[d]	ASW_Q[e]	ASW[f]
GBMLGG	0.01	1.00	1.00	0.89	0.86	0.86
BRCA	0.01	0.88	0.89	0.90	0.84	0.89
COADREAD	0.01	0.90	0.92	0.81	0.77	0.78
HNSC	0.06	1.00	1.00	0.81	0.68	0.76
LUAD	0.05	1.00	1.00	0.86	0.76	0.79
LUSC	0.05	1.00	1.00	0.91	0.76	0.87
SKCM	0.01	1.00	1.00	0.89	0.82	0.84
UCEC	0.04	0.86	0.87	0.93	0.81	0.90
ESCA	0.00	1.00	1.00	0.82	0.84	0.79
PAAD	0.17	1.00	1.00	0.79	0.64	0.73

[a] Entropy of subtype mixing.
[b] Adjusted random index.
[c] Normalised mutual information.
[d] Average silhouette width in reference data.
[e] Average silhouette width in query data.
[f] Average silhouette width in both reference and query data.

Table 5. Query and reference data overlapping evaluation in unseen test data of which distribution shifts from that of training data. Here query data are 20% test data unseen in training adding normally distributed noise and reference data are TCGA bulk tumour data.

	ESM[a]	ARI[b]	NMI[c]	ASW_R[d]	ASW_Q[e]	ASW[f]
GBMLGG	0.02	0.98	0.97	0.92	0.88	0.90
BRCA	0.02	0.94	0.94	0.91	0.86	0.89
COADREAD	0.04	0.82	0.85	0.89	0.81	0.86
HNSC	0.06	0.86	0.90	0.90	0.75	0.86
LUAD	0.12	0.71	0.73	0.62	0.52	0.60
LUSC	0.05	0.84	0.86	0.90	0.75	0.86
SKCM	0.00	0.96	0.94	0.93	0.89	0.92
UCEC	0.01	0.94	0.93	0.93	0.83	0.90
ESCA	0.00	1.00	1.00	0.80	0.80	0.76
PAAD	0.18	0.71	0.65	0.78	0.70	0.73

[a] Entropy of subtype mixing.
[b] Adjusted random index.
[c] Normalised mutual information.
[d] Average silhouette width in reference data.
[e] Average silhouette width in query data.
[f] Average silhouette width in both reference and query data.

References

1. Lotfollahi M, Naghipourfar M, Luecken MD, Khajavi M, Büttner M, Wagenstetter M, et al. Mapping single-cell data to reference atlases by transfer learning. Nature Biotechnology. 2021;doi:10.1038/s41587-021-01001-7.

2. Stein-O'Brien GL, Clark BS, Sherman T, Zibetti C, Hu Q, Sealfon R, et al. Decomposing Cell Identity for Transfer Learning across Cellular Measurements, Platforms, Tissues, and Species. Cell Systems. 2019;8(5):395–411.e8. doi:10.1016/j.cels.2019.04.004.

3. Stuart T, Butler A, Hoffman P, Hafemeister C, Papalexi E, Mauck I William M, et al. Comprehensive Integration of Single-Cell Data. Cell. 2019;177(7):1888–1902.e21. doi:10.1016/j.cell.2019.05.031.

4. Pedregosa F, Varoquaux G, Gramfort A, Michel V, Thirion B, Grisel O, et al. Scikit-Learn: Machine Learning in Python. J Mach Learn Res. 2011;12(85):2825–2830.
