# Supplementary material for: MFmap: A semi-supervised generative model matching cell lines to tumours and cancer subtypes
Source: PLoS One. 2021 Dec 16;16(12):e0261183. doi: 10.1371/journal.pone.0261183 (PMC8675718; doi:10.1371/journal.pone.0261183)
Supplement: Attachment — Submitted filename: Response_to_Reviewers.pdf [file pone.0261183.s004.pdf]

Dr. Maik Kschischo

Professor of Biomathematics

University of Applied Sciences Koblenz
RheinAhrCampus
Joseph-Rovan-Allee 2
53424 Remagen
Germany

HOCHSCHULE KOBLENZ
UNIVERSITY OF APPLIED SCIENCES
RheinAhrCampus

25 October 2021

Manuscript revision

Dear Tao Huang,

Thank you very much for editing our manuscript. We have carefully addressed your additional requirements and the points raised by you and the reviewers. Please see below our answers.

We look forward to hearing your thoughts on the matter and hope that we have overcome the criticisms. Please don't hesitate to contact me if you need any further details.

Yours faithfully, on behalf of the authors,

Maik Kschischo

Response to the Editor

1. Please ensure that your manuscript meets PLOS ONE's style requirements, including those for file naming...

We have used the PLOS Latex template and changed the naming of the files.

2. Thank you for stating the following financial disclosure...

We have added "The funders had no role in study design, data collection and analysis, decision to publish, or preparation of the manuscript." to the financial disclosure section.

3. In your Data Availability statement...
We have prepared and uploaded a minimal data set. The relevant URLS of the TCGA data and the CCLE data are provided in the Supporting Information at the end of the main text.

4. We note that you have included the phrase “data not shown” in your manuscript...

We provide the additional table as table S1 in the Supplemental file S1_file and refer to it in the main text.

5. Please upload a new copy of Figure 4, 6 and 7 as the detail is not clear...
We have uploaded new copies of these figures and hope they are clear now.

6. Please review your reference list to ensure that it is complete and correct...
We have checked the references.

Response to the Reviewer #1:
Thank you for the very positive review. Following your suggestion, we we have added data to train MFmap to the Github repo to enable other researcher to compare their work to ours.

Response to the Reviewer #2:
Thank you for the very positive review.
We have added a file S1_file.pdf as Supplemental information and refer to it in the main text. There we provide the following additional evaluations of the MFmap:

- In Table S1 we show the classification accuracy for a latent dimension of 100. You can see that the classification results are not really better, when we use a higher dimensional latent space.
- We provide additional performance evaluations for integrative analysis. We have focused on the scenario, that a reference model is trained with a large data set and a query data set (with a potential distribution shift) is fed into the reference model. We use the entropy of subtype mixing (ESM), the adjusted random index (ARI), the normalised mutual information (NMI) and the average silhouette width (ASW) mentioned in Lotfollahi et al. 2021 (https://www.nature.com/articles/s41587-021-01001-7) as performance measures for the consistency between reference and query.
- See also the sub-section "Evaluating the MFmap classification and generative performance" in the main text, were we refer to these additional performance evaluations in S1_file.pdf.
- We have cited the papers
    - https://doi.org/10.1038/s41587-021-01001-7
    - https://doi.org/10.1016/j.cels.2019.04.004
    - https://doi.org/10.1016/j.cels.2019.05.031

  suggested by you in the main text.
